# Understanding Therapeutic Experience: A Qualitative Evaluation of Virtual and In-Person Treatment

**DOI:** 10.7759/cureus.75392

**Published:** 2024-12-09

**Authors:** Brianna Cerrito, Alexa Connors, Amanda Fialk, Jamie Xiao, Frank D Buono

**Affiliations:** 1 Research and Innovation, The Dorm, New York, USA; 2 Clinical, The Dorm, New York, USA; 3 Research and Clinical, The Dorm, New York, USA; 4 Research, The Dorm, New York, USA; 5 Psychiatry, Yale School of Medicine, New Haven, USA

**Keywords:** face-to-face, online group psychotherapy, online treatment, qualitative methods, therapeutic relationship, video-delivered psychotherapy

## Abstract

Introduction

Following the COVID-19 pandemic, there was adoption of virtual psychotherapy. There are a number of benefits and drawbacks to telehealth video conferencing that are experienced by both clients and clinicians. The current qualitative study sought to outline the advantages and disadvantages that clients and clinicians have personally experienced in virtual versus in-person therapy in an effort to identify the reasons for which one medium may be preferred over another.

Methods

The research was conducted from March 20, 2023, to April 3, 2023. The interviews with 32 individuals (14 clients and 18 clinicians) took place via Zoom video conferencing. Thematic analysis of the empirical material was conducted until fit and significance were met. Application of empirical principles of qualitative research was complete when categories were saturated, and no new themes had emerged.

Results

Results indicated that virtual care is appreciated for its convenience and ease, which allows clients to attend sessions even when physical barriers limit them (i.e., sickness, vacation, and between meetings or classes). In terms of maximizing the therapeutic process, this study found that in-person care has a number of benefits that are weakened or diminished when one switches to a virtual setting. Being in-person was reported to have a significant therapeutic impact in terms of attendance, engagement, participation, understanding nonverbal communications, and developing rapport with one’s clinician and fellow clients.

Conclusion

The results of this study present an overview of client and clinician perceptions of the therapeutic impact of modes of therapy (virtual, in-person). While there is no strong evidence to suggest that one mode of care is clinically indicated over the other, the data outlines the benefits and drawbacks of both.

## Introduction

At the onset of the COVID-19 pandemic, telehealth increased by 766% during the first three months of the pandemic to protect both clients and health providers [1]. Research shows that in 2019, 0.3% of health visits were virtual, whereas the number of virtual visits in 2020 jumped to 23.6% [2,3]. These virtual visits were largely mental health appointments rather than physical health contacts, where 46.1% of all mental health sessions were conducted virtually as compared to 22.1% of physical health visits [3]. With the widespread increase in virtual psychotherapy during the pandemic, there is a growing need for research outlining the impact of virtual and in-person care [3].

Telehealth has been an effective option for clients to maintain the continuity of treatment while ensuring the health and safety of all parties involved [2,4,5]. Research has continuously demonstrated the use of telehealth visits, citing little to no significant difference from in-person sessions in terms of clinical treatment outcomes [6,7]. Additionally, virtual care provides flexibility for those living in remote environments or working in demanding careers and is associated with higher satisfaction due to lower prices and a potential increase in privacy [4,5]. Online care has also been shown to have a positive effect on client attendance, such that attendance rates for online sessions are higher than in-person sessions [8].

There are clinical barriers to virtual therapy, as a virtual setting can be impersonal, reduce levels of connection between client and clinician, and reduce the ability to pick up on nonverbal cues (i.e., body language and pauses in speech), which ultimately can have a negative effect on a clinician’s ability to build the rapport that is an integral part of psychotherapy and treatment outcomes [9,10]. Other concerns for virtual care pertain to ethical and legal questions, such as confidentiality (i.e., inability to see if others are in the room), informed consent, and potential decreased capacity to respond appropriately during the crisis due to distance between client and clinician [11]. Constant technology use can also result in burnout, as described in a 2021 study examining the relationship between fatigue and the frequency and duration of online meetings [12].

This qualitative study sought to evaluate the differences between client and clinician perceptions of virtual and in-person therapy across key therapeutic domains and whether one medium is preferred over the other. The aim of the study was to understand the impact of virtual and in-person treatment regarding key therapeutic principles. The authors hypothesized that (1) virtual care has benefits in the absence of in-person care (e.g., useful for continuity of care, distance, and financial barriers) and (2) for the safety and clinical needs of those experiencing high severity of symptoms, in-person may be preferable. In exploring these hypotheses, this paper will highlight both the benefits and potential shortcomings of telehealth from client and clinician perspectives.

## Materials and methods

Study design

This study is approved by the Institutional Review Board (IRB) at Yale School of Medicine (IRB #2000032626). The IRB has granted a waiver of HIPAA authorization for signature that allows verbal authorization for the use of PHI for the entire study. The IRB has determined that there are adequate provisions to protect the privacy of subjects and to maintain the confidentiality of data. This study is a grounded theory design, seeking to develop a theory directly based on the data collected to understand the logic of the participants’ experiences [13]. The main research question concerned the importance of whether a mode of care (i.e., virtual versus in-person) affects the therapeutic experience and alliance in mental health treatment. The statements of clinical staff and clients who had sought intensive outpatient mental health treatment since the pandemic informed about their experience. Throughout qualitative interviews, clinical staff and clients shared their perspectives on attendance, engagement, rapport-building, and the impact of acuity of mental health diagnoses when in-person versus virtual.

Data collection 

The research was conducted from March 20, 2023, to April 3, 2023. Emails were sent to staff and clients inviting them to participate in the research; thus, this was a convenience sample. Inclusion criteria were as follows: (1) must be at least 18 years of age, (2) must be currently or have been previously admitted to the Dorm or are a provider employed by the Dorm, and (3) must be able to read and understand the consent form. Exclusion criteria included the following: (1) are unfit to complete the survey due to medical or psychological constraints and (2) require a legally authorized representative (LAR). The topic of the project was presented in the emails that were sent out (i.e., the subject of the study, its course, and the purpose of the data solely for scientific purposes). The study participants were assured of the confidentiality of the study. All the respondents gave informed verbal consent in the form of a “yes” to participate in the study at the start of the focus groups. To avoid preconceptions, the interviewer tried to get to know the thoughts and feelings of the research participants by asking non-leading questions. In qualitative research, the social context of the participants’ lives, time, place, and biography are important. This was also the subject of attention both during the interview and the analytical process.

The first part of the interview guide was related to attendance and engagement in the two modes of care, and the second portion began to explore detailed topics, referring to a list of auxiliary questions, which had a semi-structured form. To obtain the best quality of data possible, the researcher asked detailed questions about experiences and reflections related to rapport-building, trust, honesty, communication, and the benefit of virtual versus in-person. The questions were open and non-leading. During the interview, all respondents were consistently encouraged to share their experiences, and much effort was made to create optimal comfort and safety in the space for this purpose.

Data analysis

The first stage involved line-by-line coding by three coders to uncover codes that were present throughout the six transcripts. The second stage focused on coding to select the most analytically significant codes to guide the search of the empirical materials. The indicated codes were considered the most important for the third stage, theoretical coding, aiming to integrate the codes generated in the focused coding stage and achieve conceptual categories. Analyses of the empirical material were completed when, in the researcher’s opinion, they met two criteria: fit and significance. In accordance with the adopted methodological assumptions, the interpretative perspective of clients and clinical staff was the leading one. There was continued application of empirical principles of qualitative research until the properties of the theoretical categories were saturated, and no new themes had emerged.

## Results

Participants

The selection of subjects was a combination of clinical staff (i.e., those with social work degrees, professional counseling degrees, nursing degrees, master of arts degrees, mental health counseling degrees, alcohol and substance use degrees, and dietetic degrees) and clients seeking intensive outpatient mental health treatment in New York, NY, and Washington, D.C. The study included 32 individuals (14 clients and 18 clinical staff members) broken into six groups of four to six individuals (three client groups and three staff groups). During the interviews, the respondents (clinicians and clients) were mostly on-site at a treatment center in New York, NY, or Washington, D.C. However, some clients were in their own homes.

At the time of the six interviews, the clients in the study were between 19 and 26 years old, with an average age of 22.29 (SD = 1.77). Of the 18 clients, 12 individuals were white (86%), one was African American (7%), and one chose not to disclose their race (7%) (see Table 1). The majority of clients were not Hispanic or Latino (n = 11, 79%), and the remainder did not disclose their ethnicity (n = 3, 21%). A total of five participants identified as cis-gender female (36%), six as cis-gender male (43%), one as non-binary (7%), one as trans-female (7%), and one as trans-male (7%). From the point of view of the analyses presented below, the distribution of these socio-demographic features remains important, as future research is warranted for diversity.

**Table 1:** Demographics table of client participants (race, ethnicity, and gender)

Demographic variables	N	Percentage
Race		
White	12	86%
African American	1	7%
Did not disclose race	1	7%
Ethnicity		
Not Hispanic or Latino	11	79%
Did not disclose ethnicity	3	21%
Gender		
Cis-gender female	5	36%
Cis-gender male	6	43%
Non-binary	1	7%
Trans-female	1	7%
Trans-male	1	7%

Clinical staff were assessed for their type of degree, which included a social work degree (n = 10, 56%), a licensed professional counselor (n = 3, 17%), nursing (n = 1, 6%), a master of arts degree (n = 1, 6%), a mental health counselor (n = 1, 6%), an alcohol and substance use counselor (n = 1, 6%), and a registered dietician (n = 1, 6%) (see Table 2).

**Table 2:** Demographics table of types of clinical staff degrees

Demographic variables	N	Percentage
Type of degree		
Social work degree	10	56%
Licensed professional counselor	3	17%
Nursing	1	6%
Master of arts degree	1	6%
Mental health counselor	1	6%
Alcohol and substance use counselor	1	6%
Registered dietician	1	6%

Thematic analysis

The in-depth interviews lasted an average of 47 minutes. As a result of six in-depth focus group interviews (three client groups and three staff groups of four to eight individuals per group), recorded audio material of four hours 40 minutes was obtained, which was then subjected to naturalized transcription (a copy of the spoken discourse). Because the collected research material turned out to be extremely rich in important data, there was no need to conduct repeat interviews.

**Figure 1:**
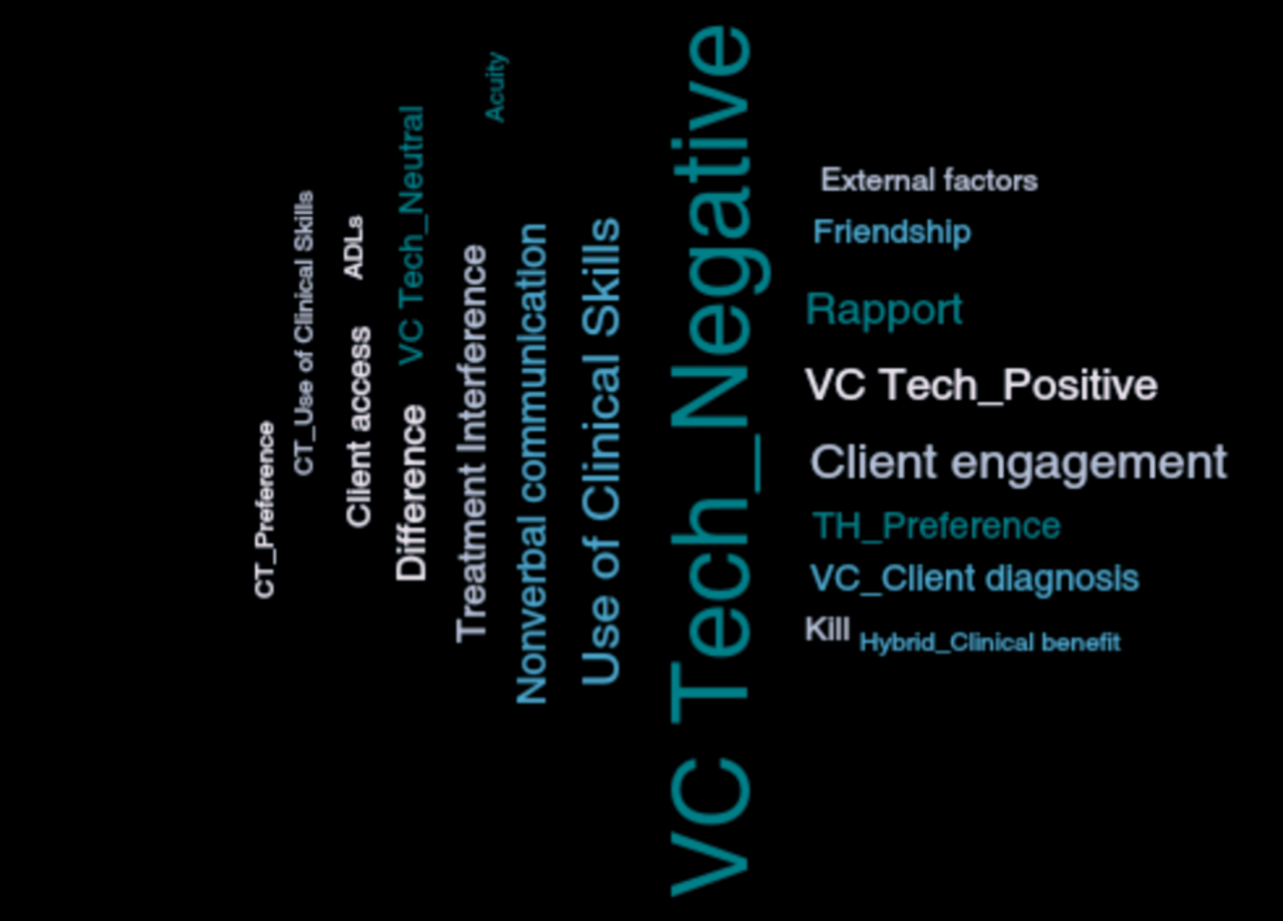
Code cloud visual displaying codes relative to their frequency of use in transcripts

Following thematic analysis, a total of 11 theme codes were collapsed into three thematic categories (see Table 3). The codes of greatest analytical importance that occurred most frequently were virtual care (technology), client engagement, nonverbal communication, use of clinical skills, and rapport (see Figure 1). 

**Table 3:** Number of times each code was applied to transcripts in analysis

Themes	Codes	Frequency of application
Issues in virtual care	Negative impact of technology	172
	Treatment interference	38
	Nonverbal communication	44
	Use of clinical skills (clinician perspective)	64
	Use of clinical skills (client perspective)	11
Benefits of virtual care	Positive impact of technology	52
	Client access	28
	External factors	26
	Client attendance	37
Therapy mode preference	Client preference	10
	Clinician preference	36

Issues in virtual care

Clients reported concerns about distractions and body language that result from receiving individual and group therapy sessions through a screen. They find that there are barriers to being fully attentive in treatment (i.e., checking notifications and the temptation to look at another tab) and concern that the clinician could miss important information when assessing them through a screen. Clinicians struggled with concerns around their clients’ focus and attention, the ability to maintain maladaptive patterns if doing therapy in their home environments, and difficulty reading body language through a screen.

Four sub-themes were identified under the issues in virtual care: negative impact of virtual care, treatment interference, nonverbal communication, and use of clinical skills.

Negative Impact of Technology

First, the negative impact of virtual was defined as any mention of using technology for mental health treatment that is negative. Client feedback supported in-person as a better option for those who are experiencing acute symptomatology or in situations that would interfere with the ability to get the most out of treatment. Clinicians feel that clients can maximize the benefits of treatment when in-person due to decreased distractions, and they report that utilizing clinical skills, as well as picking up on cues, are much more effective when in person. The below examples evidence this:

“I think engagement is there, but focus and attention gets impacted. Like even if my client brings something into session and we’re talking about it, if middle of the session they get an alert or an email from their work…” (Staff Group #3)

“Respondent #5: We’ve gotten to our ideal. Like in person, no mask. We can see it all, and we have 100% of the information available to us in that moment. So yeah, it’s definitely just, was the last barrier down in terms of, like, physical body language and stuff. Respondent #3: Yeah, I agree.” (Staff Group #2)

“Even things like your ADLs. You can just walk past a client and smell whether or not this person took a shower or whatever. You could just see in a snapshot if they had a long night or whatever the situation is just walking around the place, seeing how they either get somethin’ to eat, how they socialize with other people. Even before you even start the group and they sit down, their body language with that. You can hear tonality a lot differently when you’re interacting with someone.” (Staff Group #1)

Treatment Interference

In the case of treatment interference, the features of virtual technology make it difficult to pick up on patterns that would help to inform treatment. For example, in the case of an alcohol use disorder, you could smell if a client is using alcohol when in-person, but this might be missed through a screen. Additionally, there is concern around safety, as those who are struggling with acute mental health issues like domestic abuse or substance use could be in imminent danger by remaining in the triggering environment. These clients may need to physically leave the home to obtain a benefit from treatment. The below statements evidence these analyses:

“…people can discretely use substances and maintain use patterns…I think being in person and like seeing someone walk and sit down and being with them in-person is really helpful…there’s just…an added like ability to, I think, go unnoticed and for folks to evade detection or toxicologies or any sort of, anything that will like get them found out. I think that’s probably similar with eating disorders. We’ve seen people just completely refuse to like show how much they’ve eaten, or they just deny that they like went to the restroom right after eating.” (Staff Group #3)

“…I was in a really abusive and codependent relationship. And so when I was in virtual care then, I think it would have been more helpful to be in in-person care because I needed to be, I was in this person’s house quietly talking about them, right, and then just going right back to that environment that was super unhealthy…I needed some distance from my environment because of where I was in my process.” (Client Group #2)

The respondents admitted to a distractibility that occurs from using virtual technology in session. Clients and clinicians reported feeling that it was easier to text, check emails, and surf the web while in session, whereas multi-tasking likely wouldn’t take place in an in-person setting. The below statement is an example:

“I found that in virtual sessions, while they’re still useful, there’s inevitably going to be implicit distractions. Even if you’re not explicitly trying to surf the web, you see your notifications everywhere. There’s a top clock in the corner of your screen. And impulsively, sometimes I feel like I just click my email and I go back, but I think that takes away from the interpersonal experience.” (Client Group #1)

Nonverbal Communication

Clients and clinicians report that virtual care can hinder the ability to read body language, facial nuances, and visual cues. As a result, the therapeutic alliance may be weakened. The below statement summarizes the sentiment across clients and clinicians:

“The way that I present myself does come a lot through body language through posture, through being able to kind of express myself physically and with my voice and with what I’m saying, so I feel like a large part of what could create some sort of therapeutic alliance is taken down because, at the end of the day, we’re lookin’ at screens instead of each other.” (Staff Group #1)

“I will say a huge portion of the work that I’ve been doing…is trauma related. And so…on the body language front, one downside to virtual care is that it’s a lot easier for me to lie, basically, not lie but avoid topics that make me uncomfortable. Whereas in-person, it’s harder to…get my entire body language to not…telegraph that there is something that I don’t want to talk about or feel ashamed about and shame being such a huge portion of trauma work. In that way, I think being in-person has been helpful for that element of therapy.” (Client Group #2)

Use of Clinical Skills (Clinician and Client Perspective)

There was concern expressed about the ability of clinicians to use their clinical skills as they translate into a virtual medium. For example, clinicians feel that using a screen prevents them from being able to track ADLs since there is a loss of all five senses through a screen (e.g., smelling alcohol or noticing that someone hasn’t showered). The below excerpts outline examples of this hindrance:

“I feel like when you’re online, it takes a lot of your assessment skills away in terms of a clinician. Even things like your ADLs.” (Staff Group #1)

“I think that on Zoom or virtually, it’s harder to address engagement or lack of engagement, so that’s, I think, where it becomes a little different because, yeah, it’s very easy for someone to shut off their video even though we ask them not to. But it’s hard for us to sort of pull them back out, whereas, in person, it’s a lot easier to ask them a question and look them in the eye and have them know we’re talking to them and sort of feel the pressure if you will, the group pressure or groupthink process to get them involved.” (Staff Group #2)

“like the intricacies, the nuances of facial expressions and body language in therapy…being able to see how someone is physically reacting to what you’re saying is important. And I feel like also for the therapist maybe I’ve - all of a sudden, I’m uncomfortable, there might be a physical tell that they might be able to pick up on.” (Client Group #2)

The above statements, reported by both clients and clinicians, suggest that using virtual mediums of therapy may hinder the use of clinical skills by the mental health provider. This is due to the inability to read body language, notice intricate facial expressions, or hear variations in tonality that would otherwise be noticeable when interacting in-person. There are also features native to virtual technology that allow clients to turn off their cameras or mute themselves, which creates a further barrier to being able to read a client. This leaves only the opportunity for an “educated guess,” which is not an informed clinical observation.

Benefits of virtual care

Clients report that virtual treatment is a helpful option for continuity of care, especially when external factors (i.e., work, school, sickness, and vacation) get in the way. Clinicians also referred to virtual care as a good “backup” in allowing for flexibility in times of need, as opposed to no therapy at all. However, clients did admit to higher attendance when in-person, as “it’s easier to just not open your computer than blow someone off in-person.”

Four sub-themes were identified under the benefits of virtual care theme: client access to care, client attendance, external factors, and positive impact of technology.

Client Access

Client access to care was defined by the ease and ability of a client to access virtual care. Particularly in the client groups, participants noted the convenience of being able to log onto virtual care from any location during a busy school or workday or even a vacation, which may at times serve as a hindrance to being able to show up on time or at all to appointments. Other clients noted the financial barriers that arise in getting to and from appointments and that virtual care provides more accessibility to those with different economic backgrounds. Several clients also compared the physical ease that it takes to log onto a virtual session versus the amount of effort it takes to attend a session in person.

“I think obviously it makes attendance feasible for some people that physically cannot get to the places, so I guess in a way that attendance does get better when it is virtual for people who, due to financial or physical barriers, can’t get to a scheduled appointment on time.” (Staff Group #1)

“This includes people that are incredibly busy. And like if somebody’s like, ‘I only have one hour in my day, and half an hour of that would be spent traveling to an office versus I can just open up my laptop,’ I think just the fact that it became so much more, therapy has become so much accessible and feasible to people of many different backgrounds. I think that’s definitely the saving grace of virtual care.” (Staff Group #1)

“It’s just easier to show up. It’s less of a commitment. You don’t have to get dressed, put shoes on, go outside, take the train, all that stuff, think about transit time. It’s easier to just click a link and just be done, you’re there already.” (Client Group #1)

Client Attendance

The sub-theme of client attendance referred to the ways in which virtual care has impacted attendance in both individual and group therapy sessions. Some felt that because care was easier to access from anywhere, it was easier to schedule appointments during times they may not otherwise be able to. Without virtual accessibility, care may not have been an option at all.

"It’s, I would say, more beneficial to be in-person. However, when I go back to school, I’m going to be busy, and there’s going to be times where I want to get a session in, and I wouldn’t be able to come in-person just because my day is hectic, but I’d be able to schedule something online. So I would say the benefit is just the accessibility and the ability to squeeze in an appointment you might otherwise have not had.” (Client Group #1)

However, other clients shared that they felt more accountability around in-person individual appointments and, therefore, were less likely to cancel those appointments. Similar to how participants felt the ease of a simple click to log onto an appointment, some participants felt a similar ease of canceling virtual appointments.

“I’d say that I attend in-person sessions around 90%, 95% of the time, and I would say that that number is around closer to 70% to 75% when I am virtual.” (Client Group #2)

“There are times where I feel like it is easier to cancel when it’s online as opposed to in-person because you’re not just showing up a person. It’s easier to just not open your computer than blow someone off in-person.” (Client Group #1)

External Factors

The sub-theme external factors referred to legitimate barriers to obtaining treatment in-person. Participants often referred to situations like traffic, being sick, meetings going over-time, and busy schedules that make it difficult to show up in person on a regular basis. Though participants often referred to the value of in-person sessions, they liked the virtual option as a backup when in-person was not possible.

“I really appreciate the moment, the times when my schedule isn’t going to work unless I do it virtually. I had, last Wednesday, we had a group, and I had to go to Williamsburg afterward, and I did not have time to make it from here to Williamsburg, but I did have time to make it to my apartment there. So that was really helpful, and I was still able to benefit from that group even though I didn’t really say anything because speaking virtually is awkward.” (Client Group #2)

“It’s a good backup in that sense where it’s like, ‘I literally can’t come in’ for whatever reason, whether it’s medical, physical, emotional.” (Staff Group #3)

Clinicians too spoke to the ease at which they could make appointments for clients around their busy school and work schedules. For clients who were more highly acute and needed more oversight, in-person would remain a first choice; however, for clients who did not and were transitioning into fuller lives outside of treatment, virtual was a good option.

“Because what I find out is like when you’re getting your schedules together for your clients, they’ll have like a class on like Tuesday, Thursday and on Monday, they get off at this particular time. It gives you a lot more flexibility in those situations. When you have, like, the tier 1’s, where they’re here all day, the benefit is not as strong. But a lot of the people that are transitioning, tryin’ to get a job, doin’ jobs outside, or in school, there’s a lot more flexibility when it comes to the virtual thing.” (Staff Group #1)

“Yeah, I think just if like one-off situations come up. Like I had a client need to change…and it was usually in person but this one time just wanted to do Zoom, so being able to do that without like, ‘No, you have to be on vacation or be sick to be virtual,’ and knowing maybe on a case-by-case basis…I think it is largely more effective to be in person like everyone else has said too, but to have the flexibility and option can be nice and maybe encourage more participation if someone doesn’t feel like, ‘Oh, I have to be in person no matter what even if X, Y, Z comes up.’” (Staff Group #1)

Positive Impact of Technology

The sub-theme positive impact of technology referred to the mention of ways in which technology has positively influenced the therapeutic process. For clients in their first stages of treatment, in-person care would be a first choice due to needing more oversight, more symptomatic, and more acute. However, the use of virtual care became particularly valuable as clients moved through the treatment process and got back to their lives outside of treatment. Virtual care allows clients to balance their therapeutic and nontherapeutic lives.

“And I think that once I started, things started getting back to normal, whatever that means, being less immediate intensive, unsafe, I think that’s when it became a lot more helpful for me to have virtual care. Because at that point, I was just really busy, and it became more stressful than not for me to come in-person, and the benefits were pretty minimal at that point relative to virtual care. So I think that it’s very individual-based, and it depends on where people are at in their journey.” (Client Group #2)

Virtual care also opened a window for clinicians to see into clients’ lives in ways they would not have been able to via in-person sessions. Sessions could take place in any setting (i.e., bedrooms, offices, or coffee shops), which at times served as conversation starters and a way to build rapport between clients and clinicians and even led to an increase in client comfort, allowing them to open up more readily due to being in a familiar space.

“I’ve definitely had some clients feel like it’s been easier to open up virtually in individual sessions maybe, because maybe it’s more difficult in person, just like the atmosphere is more intimidating.” (Staff Group #2)

“To be, play the devil’s advocate of like what [clinician] was saying, in a way, though, because they’re more comfortable in their bedroom and they’re having different behavior, there could be that extra opening or opportunity to see some things that we might not get to see in a therapy setting that might progress the work that we’re doing with them.” (Staff Group #2)

Therapy mode preference

Client and clinician feedback indicated favorability for in-person care as the more effective treatment option, especially when considering high-acuity mental health diagnoses. Two sub-themes were identified regarding the perceived preference for mode of therapy: client perception and clinician perception. Most clients and clinicians reported a perspective that in-person care, especially in terms of clinical gain, is more effective than virtual care. It is important to note that though this sentiment was shared by most clients and clinicians, some clinicians reported a preference for virtual care due to its convenience.

Client Preference

Overall, clients reported that in-person treatment offers greater engagement in the session, a larger connection to the community, and, overall, a more enhanced therapeutic experience rather than attending virtually. One client noted the following about in-person care:

“feels a lot more potent overall when you’re in the space talking. Yeah, it just feels a lot more potent.” Another participant added that during virtual sessions, it is “difficult to focus because it’s virtual….And then as far as groups go…they are more engaged in person than virtually.” (Client Group #3)

Likewise, clients reported a stronger connection to the treatment community when they were involved in in-person versus virtual care:

“I think there is a benefit of going to the dorm in-person for sessions because it builds a sense of community, which personally is something I was really looking for when I came to the dorm.” (Client Group #3)

“There’s a community here where there’s just more accessibility to help when it’s just not on Zoom because it’s way harder to call someone or dial them to get help as opposed to just walking up to them and asking them a question. I’m talking about clinicians as well as fellow clients. So absolutely, I think there is an immense benefit to going to the dorm.” (Client Group #1)

“Benefit to seeing other people in the same place as you physically and in the same place as you metaphorically.. it’s just comforting. It makes me feel less self-conscious to be part of a physical community of people who are struggling with similar things. It just is a little bit less isolating than sitting just by yourself in a room.” (Client Group #1)

Clinician Preference

Most clinicians who participated in the study preferred in-person versus virtual care, identifying rapport/connection, body language/nonverbals, and effective treatment of specific diagnoses as reasons for their preference. The below statements outline the strong preference toward in-person care:

“I’m in-person. I hate Zoom in every iteration that it comes in. I hate it. I tolerate it because I have to.” (Staff Group #3)

“I feel like it’s harder virtually to treat anyone.” (Staff Group #2)

Many clinicians noted that building rapport in person is easier with in-person contact than being on a screen. The below statements evidence this perspective that in-person therapy is more effective:

“I prefer to meet someone in person to build a rapport versus starting doin’ any Zoom session.” (Staff Group #2)

“Zoom or virtual services was like kind of the equivalent of a long-distance relationship. And I’ve told so many clients that when they are in a long-distance relationship and I have told them, I said, No one gets into a relationship and says, ‘You know what would make this better? Let’s do it long distance,’ that it’s something they have to kind of navigate and kind of get through. But I have to rethink that because that’s how I thought about virtual, right? It’s like it’s not ideal. No one gets into therapy saying, ‘You know what I wanna do? I wanna get into therapy, but I wanna do it virtually. That’s gonna make it better.’ But again, I’m having to rethink that paradigm a little bit.” (Staff Group #2)

Many of the clinicians reported that there are certain diagnoses and symptoms where virtual care is not only less preferred but contraindicated for the sake of treatment:

“Virtually, my clients who focus more on executive functioning, those ones work out more, but the ones with the eating disorder, they have to be in person for weights and other things like that. Like they can hide if they’re eating or not eating better when they don't see me in person.” (Staff Group #3)

“acute schizophrenic or schizoaffective, no, that won’t work, as well as someone that’s, I mean, ADHD, something you can’t say acute or not acute. It’s like where are they at in that particular phase with themselves at that time. And, of course, those who are acutely suicidal or self-harm, that’s a no-go.” (Staff Group #2)

“I hear you, but if you’re workin’ with someone that’s, have sexual trauma, I mean, I wouldn’t want be doin’ a session with them while they’re in their bedroom. Just like certain pathologies, really, you wanna help them not get triggered or PTSD, I think, or, I mean, I just notice that as a therapist, I mean, I’m not comfortable with them doing that because I think, again, they need to have more of a different setting than that type of intimate setting.” (Staff Group #2)

Clinicians also pointed out that it is easier to pick up and read body language and nonverbal communication when in-person versus virtual with clients.

“We’ve gotten to our ideal. Like in person, no mask. We can see it all, and we have 100% of the information available to us in that moment. So yeah, it’s definitely just, was the last barrier down in terms of like physical body language and stuff.” (Staff Group #2)

Some clinicians in the study did prefer a virtual care model pointing to the convenience of being able to see clients virtually.

“Sometimes it’s just kind of a relief, a little more relaxing, at least in my experience, to just be virtual once in a while.” (Staff Group #1)

“Now I do know that if there are times when I’m feeling not quite as invested or motivated in the particular day or hour, it might be nice to, like, ‘Oh, okay, we’ll do it virtually,’ and it can be an easier hour in a way.” (Staff Group #2)

## Discussion

The present study sought to understand whether virtual care is useful for continuity of care in the absence of being able to physically attend treatment (e.g., distance, financial barriers) and whether in-person care is an important factor for high acuity clients in terms of engagement, safety, and clinical benefit. In terms of virtual care having the added barrier of a screen, there is an inherent ease of reading body language and nonverbal cues that may not happen as easily without being able to see the person’s whole body. This also includes assessment of activities of daily living (ADLs) (e.g., whether a client has showered, brushed their teeth, and dressed appropriately for the day), which is significantly harder without the full capability of one’s senses, such as the ability to smell. Staff and clients both expressed concern for virtual treatment impairing the use of clinical skills that are essential to the treatment process. Prior research shows that clinicians conducting virtual therapy perceived poor outcomes, diminished use of therapeutic skills, and reduced therapeutic influence when conducting teletherapy compared to in-person therapy [14]. Clients in the present study also admitted to the temptation of checking notifications or browsing the web when doing therapy virtually. This poses a treatment-interfering factor, potentially weakening the impact of the therapeutic relationship due to lack of presence.

Our findings on virtual care are consistent with prior research, which has found that clients of virtual therapy report advantages such as convenience, easy scheduling, and lack of travel time [15,16]. The aspects of convenience and ease were noted because virtual sessions allow clients to attend even when sick or on vacation. It was also suggested that having the option to be virtual in case-by-case situations is helpful, as doing therapy virtually is better than not attending therapy at all. Especially when individuals are doing well (e.g., working, in school, and managing mental health and well-being), there comes a point where virtual care is thought to be appropriate for the sake of continuity. Our findings also suggest that doing therapy from one’s home, in certain contexts, may encourage the individual to open up and be more vulnerable. While there was more admitted accountability to show up when in-person, virtual care was appreciated for its utility in making it easy to fit therapy within a busy schedule.

The overall shared perspective is that in-person treatment is most effective, especially for high-acuity mental health disorders. It was felt that, in terms of gaining the most benefit from treatment, there is much more accessibility to the clinician and fellow peers when clients are on site. For the clients, being in-person makes it easier to ask for help or seek additional support, whereas, on the clinician's side, it is easier for the clinician to access the client. The general agreement was that rapport building and engagement in the treatment process and broader milieu are optimal when in-person with others. While the therapeutic alliance is certainly possible in both settings, alliance building is harder in a tele-mental health setting (i.e., relying on what they can see of the clients’ environments, tone of voice, and facial expressions) [17]. Concern was expressed by both clients and clinicians; however, consistent with prior research, clinicians show greater concern about the therapeutic alliance in virtual settings than clients do [18].

Implications

The empirical material collected from in-depth focus group interviews with clinical staff and clients allowed us to document the conditions of therapeutic interactions in virtual and in-person mediums. Published literature has suggested that virtual care provides flexibility and higher satisfaction in terms of ease of access to care [4,5]. Our results support this and further explain that virtual care is deemed useful for its convenience and ease because it allows clients to attend sessions regardless of circumstances (i.e., sick, on vacation, and at work), and there is more admitted accountability to show up and engage when sessions are in-person. In terms of maximizing the potential for a therapeutic alliance, our transcripts revealed a preference for in-person. Participants explained that virtual settings could reduce connectedness between client, clinician, and members of group therapy sessions, as well as hinder the ability to pick up on nonverbal cues (i.e., body language and pauses in speech), further impacting the clinician’s ability to build the rapport, and this is consistent with existing literature [9,10]. It can be understood from our results that virtual and in-person treatment can both be effective in different ways. In-person can be considered better for maximizing the therapeutic experience and alliance, especially for high-acuity mental health disorders who need a higher degree of oversight and clinical attentiveness.

Limitations

The analyzed group of clients and staff is not representative of the broader community of mental health professionals and young adults seeking intensive outpatient mental health treatment. Our results may not sufficiently reflect the experiences of individuals with high-acuity mental illness seeking intensive outpatient treatment or clinicians who treat this population. Thus, we cannot be certain whether the results of this study would generalize to the broader population. The research attempted to help in understanding the differences between modes of care (virtual, in-person), considering the individual and contextual differences that might qualify these experiences. We are aware that these results represent a specific group of individuals studied at a specific time (i.e., post-COVID-19 pandemic), from large urban settings (i.e., New York, NY, and Washington, D.C.) and social context (i.e., predominantly high socioeconomic status); therefore, future research is warranted to further explore this concept in diverse populations.

## Conclusions

The results of the reported qualitative study present an overall picture of how clients and clinicians perceive the therapeutic impact of modes of therapy (virtual, in-person). While there is no strong evidence to suggest that one mode of care is clinically indicated over the other, the data outlines the benefits and drawbacks of both. However, in terms of maximizing the therapeutic process, this report has shown that in-person care has several benefits that are weakened or diminished when one switches to a virtual setting. Thus, virtual care is understood to be ideal for continuity of care when external barriers would otherwise prevent treatment and in cases where clients are fully stable, and symptoms are manageable. Being in-person was reported to have the most significant therapeutic impact in terms of engagement, participation, attendance, understanding nonverbal communications, and developing rapport with the clinician and fellow clients. Both modes of care have a place and benefit from client and clinician perspectives, with a consensus that in-person is perceived as more clinically beneficial in most cases.
